# Cognitive frailty in patients with acute coronary syndromes: A scoping review

**DOI:** 10.1371/journal.pone.0361499

**Published:** 2026-10-30

**Authors:** Min He, Yifan Wu, Mengting Zhang, Yuan Chu, Jinbo Zhao, Zhiping Liu, Yongchao Hou

**Affiliations:** 1 School of Nursing, Shanxi University of Chinese Medicine, Jinzhong, Shanxi, China; 2 Department of Central Laboratory, Shanxi Province Cancer Hospital, Taiyuan, Shanxi, China; 3 Jining No.1 People’s Hospital, Jining, Shandong, China; 4 Shanxi Province Academy of Traditional Chinese Medicine, Taiyuan, Shanxi, China; 5 Emergency Department, Shanxi Provincial People’s Hospital, Taiyuan, Shanxi, China; Gabriele d’Annunzio University of Chieti and Pescara: Universita degli Studi Gabriele d’Annunzio Chieti Pescara, ITALY

## Abstract

**Aims:**

To address a critical knowledge gap, this is the first scoping review to systematically map and describe the evidence on cognitive frailty in patients with acute coronary syndromes (ACS), including its prevalence, assessment tools, associated factors, and clinical outcomes.

**Methods:**

The Joanna Briggs Institute methodology guided the conduct of this scoping review, which has been reported in line with the PRISMA-ScR checklist. Following the PCC framework, a systematic search was conducted across five databases: PubMed, Embase, CINAHL, Scopus, and Web of Science. The search was performed up to August 2026. Two reviewers independently screened studies, extracted data, and assessed quality.

**Results:**

A total of 9 studies were included. The prevalence of cognitive frailty ranged from 8% to 55%, the differences attributed to the heterogeneity of assessment tools and diagnostic criteria. Regarding assessment tools, the FRAIL scale combined with the Short Portable Mental Status Questionnaire was the most commonly used combinations. Risk factors covered sociodemographic, physical health, and clinical domains. Prognostically, cognitive frailty was linked to higher composite short‑term (in‑hospital complications, prolonged stay, 30‑day events) and medium‑term (6‑month MACCE) risks, and also to elevated long‑term mortality and readmission.

**Conclusion:**

Cognitive frailty is commonly reported in patients with ACS, with considerable variation across studies, and an association with adverse outcomes has been noted in multiple reports. However, the lack of standardized assessment tools and criteria makes results difficult to compare and synthesize. Existing prospective studies have primarily relied on baseline measurements of cognitive frailty to predict subsequent outcomes, without repeated assessments over time. This limits the ability to characterise the dynamic trajectory of cognitive frailty during the ACS course. Future research should focus on longitudinal studies, the development of rapid screening tools, and the exploration of multidisciplinary interventions.

## 1. Introduction

Cardiovascular disease (CVD) is a major health burden and the leading cause of death, nearly half of these deaths attributed to ischemic heart disease [1]. Acute coronary syndromes (ACS) is a common and severe form of ischemic heart disease, including ST-Segment elevation myocardial infarction (STEMI), Non-ST-Segment elevation myocardial infarction (NSTEMI), and unstable angina (UA) [2]. Unstable angina and non-ST segment elevation myocardial infarction are often continuous and clinically indistinguishable, collectively referred as non-ST elevation ACS (NSTE-ACS) [3]. ACS is characterized not only by a high mortality rate during the acute phase, but also by poor prognosis for survivors, with a significantly increased risk of recurrent cardiovascular events and heart failure [4,5].The long-term prognosis of ACS survivors is shaped not only by the initial cardiac event but also by subsequent physiological processes. Persistent inflammation, coagulation activation, and neuroendocrine alterations create a milieu that predisposes patients to physiological and cognitive impairments [6]. Consequently, these factors further impair health resilience and quality of life [7,8], compounding the overall prognostic burden.

Cognitive frailty, as a new concept that includes both physical and cognitive dimensions, has become an increasingly prominent concern in cardiovascular diseases. In their 2013 consensus statement, the International Academy of Nutrition and Aging (IANA) and the International Association of Gerontology and Geriatrics (IAGG) proposed a definition for cognitive frailty and established its diagnostic criteria. cognitive frailty as the co-existence of physical frailty and cognitive impairment, while explicitly ruling out Alzheimer’s disease and other dementia types [9]. Ruan et al. refined the definition of cognitive frailty in 2015 [10]. They proposed that cognitive impairment component of cognitive frailty resulted from physical or prephysical frailty and comprised two subtypes: reversible and potentially reversible. Here, frailty is defined as a decline across multiple physiological systems, leading to reduced reserves and diminished resistance to stressors [11]. Among patients with ACS, the prevalence of frailty can be as high as 82.4% [12]. Frailty leads to poor short-term and long-term clinical outcomes, and is also associated with a significant deterioration in health-related quality of life [12,13]. Mild cognitive impairment may be a precursor to dementia, is highly prevalent among post-ACS patients, reported rates ranging from 15.1 to 52% [14]. It compromises patient adherence to secondary prevention and self-management [15], thereby directly contributing to adverse clinical outcomes [7].

Evidence from cross-sectional and longitudinal studies demonstrates a significant association between these two conditions [16,17], either of these two geriatric syndromes can increase the risk of developing the other syndrome. It has long been debated that frailty measures underestimate other domains of frailty, including cognitive function. Consequently, integrating cognitive function with frailty measures has led to the conceptualization of cognitive frailty. More importantly, cognitive frailty is associated with a greater risk of disability, hospitalization, and mortality compared to either condition alone [18]. Cognitive frailty poses a significant challenge to the prognosis of survivors of ACS and is a key clinical indicator for predicting adverse outcomes in this population.

Cognitive frailty is considered a potentially reversible condition [10], and early identification and intervention may improve outcomes and quality of life in patients with ACS. To our knowledge, no previous scoping review has systematically mapped the available evidence on cognitive frailty specifically among patients with ACS. Scoping review methodology offers distinct advantages in systematically mapping existing evidence on a topic, providing a comprehensive overview of the research landscape, identifying knowledge gaps, and compared to traditional literature reviews, this method provides a more structured and transparent way to examine existing work. Given these strengths, we selected the scoping review method for our study. This allowed us to describe and critically assess the literature relevant to our research focus [19]. This review aims to systematically synthesize the evidence on cognitive frailty in patients with ACS, focusing on its prevalence, assessment tools, influencing factors and outcomes, thereby providing scientific evidence to support early screening, preventive strategies, and quality-of-life improvements for ACS patients with cognitive frailty.

## 2. The study

### 2.1 Aim

To systematically map and describe evidence on cognitive frailty in patients with ACS and its implications for prevalence, assessment tools, influencing factors and outcomes.

### 2.2 Research questions

We used the Population, Concept, and Context (PCC) framework [20] to make the research questions for this scoping review. The Population (P) was patients with ACS. The Concept (C) was cognitive frailty. This clinical syndrome is defined by the simultaneous presence of physical frailty alongside cognitive impairment, and it does not include Alzheimer’s disease and other dementias. The Context (C) was different care places, like hospitals, community centers, and homes. The overarching research question guiding this review was: What evidence is available on cognitive frailty in patients with acute coronary syndrome? To address this, four specific sub-questions were formulated: (1) How common is cognitive frailty in patients with ACS? (2) What clinical instruments are used to evaluate cognitive frailty in this population? (3) Which factors are associated with the development of cognitive frailty in ACS patients? (4) How does cognitive frailty change health results for people with ACS?

## 3. Methods

### 3.1 Design

This review adhered to the Joanna Briggs Institute (JBI) methodological framework [21] in its design and conduct, which builds upon the original work by Arksey and O’Malley [22]. A systematic literature search was performed across multiple electronic databases to identify relevant studies. After completing the data extraction, a quality assessment method was used to verify the reliability of the data. The entire review process was independently carried out by two researchers, with any disagreements resolved through discussion or consultation with a third-party reviewer. The study was registered prospectively on the Open Science Framework (https://doi.org/10.17605/OSF.IO/M7QDF).

### 3.2 Inclusion and exclusion criteria

The inclusion and exclusion criteria were established in accordance with the PCC approach. Table 1 details the corresponding criteria pertaining to the population, concept, context, and types of evidence sources.

**Table 1:** Inclusion and exclusion criteria.

PCC	Inclusion criteria	Exclusion criteria
Population	Studies that include adults 19 years or older, of any culture, sex, or ethnicity, who have been diagnosed with Acute Coronary Syndromes, are eligible for inclusion.	
Concept	Cognitive frailty must be defined as physical frailty/prefrailty and mild cognitive impairment simultaneously. Physical frailty needs to be identified by internationally recognized diagnostic criteria (e.g., Frailty Phenotype, Frail Scale or others). Cognitive impairment needs to be defined according to established cognitive screening measures widely used worldwide, such as the Mini Mental State Examination, Clinical Dementia Rating or others.	
Context	Studies conducted in all kinds of settings, including home, community, rehabilitation, hospital, facility, residential home, and nursing home, are included.	
Types of Evidence	• Qualitative studies (e.g., interviews, focus groups) • Quantitative studies (e.g., surveys, observational studies) • Mixed-methods studies • Grey literature (e.g., government reports, policy documents, competency frameworks)	• Studies were excluded, including Literature reviews or scoping/systematic reviews, opinion pieces, viewpoints, review protocols, conference abstracts, commentaries, conference proceedings/ abstracts, and book reviews. • Studies without full text

### 3.3 Search strategy

Based on the JBI guidelines [21],the PCC formula was used to define the main concepts. Additionally, the Peer Review of Electronic Search Strategies (PRESS) framework [23] was adopted to guide the development of the search strategy. This review searched five major databases: PubMed, Embase, CINAHL, Scopus, and Web of Science. The search covered the period from database inception to August 13, 2026, and combined Medical Subject Headings with free text terms. Detailed search strategies for the five databases are provided in Table 2, the detailed search results can be found in S2 Table. While the JBI methodology advocates for inclusion of all languages, practical limitations led to the exclusion of non-English publications in this review.

**Table 2:** Search terms and strategy.

Concepts	Keyword	Subject heading (index)
**Acute Coronary Syndrome**	“Acute Coronary Syndromes” OR “heart attack” OR “Coronary Syndrome*, Acute” OR “Syndromes, Acute Coronary” OR “ACS” OR “ST Elevat* Myocardial Infarction” OR “ST Segment Elevation Myocardial Infarction” OR “STEMI” OR “Non-ST Elevated Myocardial Infarction” OR “Non ST Elevat* Myocardial Infarction” OR “NSTEMI” OR “Non-ST-Elevation Myocardial Infarction*” OR “Infarction*, Non-ST-Elevation Myocardial” OR “Myocardial Infarction*, Non-ST-Elevation” OR “Angina*, Unstable” OR “Unstable Angina*” OR “Angina at Rest” OR “Angina*, Preinfarction” OR “Preinfarction Angina*” OR “Angina Pectori*, Unstable” OR “Unstable Angina Pectori*” OR “Myocardial Pre-infarction Syndrome*” OR “Pre-infarction Syndrome*, Myocardial” OR “Syndrome*, Myocardial Pre-infarction” OR “Myocardial Infarction*” OR “Infarction*, Myocardial” OR “Heart Attack*” OR “Myocardial Infarct*” OR “Infarct*, Myocardial” OR “Cardiovascular Stroke*” OR “Stroke*, Cardiovascular” OR “Ischemias, Myocardial” OR “Ischemic Heart Disease” OR “Diseases, Ischemic Heart” OR “Artery Disease, Coronary” OR “Artery Diseases, Coronary”	**PubMed:** “Acute Coronary Syndrome”[Mesh] **Embase:** ‘acute coronary syndrome’/exp **CINAHL Complete:** MH “Acute Coronary Syndrome” **Scopus:** no index term[ti/ab] **Web of sciences:** no index term[ti/ab]
**Cognitive frailty**	“Cognit* frail*” OR ((frail* OR “Frailty Syndrome” OR “physical frailty”) AND (Cogniti* OR “mild cognitive impairment*” OR “Cognitive Function*” OR “Function*, Cognitive” OR “Disorder*, Cognition” OR “mci” OR “cognitive decline*” OR “cognitive impairment*” OR “Cognition Disorder*” OR “Cognitive Dysfunctions” OR “Dysfunction, Cognitive” OR “Dysfunctions, Cognitive” OR “Cognitive Disorder*” OR “Impairment*, Cognitive” OR “Cognitive Impairment*, Mild” OR “Impairment*, Mild Cognitive” OR “Decline*, Cognitive” OR “cognitive defects” OR “cognitive deficiency” OR “cognitive deficit” OR “cognitive difficulties” OR “cognitive disability” OR “cognitive problems”))	**PubMed:** no index term **Embase:** no index term **CINAHL Complete:** no index term **Scopus:** no index term[ti/ab] **Web of sciences:** no index term[ti/ab]

### 3.4 Search outcome

All identified records were imported into Covidence for management and deduplication. After duplicate removal, two reviewers examined the titles and abstracts of every remaining record independently, applying the predetermined selection criteria. Articles judged as potentially relevant were then obtained in full text and subjected to independent evaluation by the same two reviewers to reach a final decision on inclusion. Any disagreements between the reviewers regarding eligibility were resolved through consensus discussion or, when needed, by adjudication from a third reviewer.

A total of 1671 records were initially retrieved from the database searches. Following the removal of duplicate entries, 1050 unique records remained for screening. After two consecutive rounds of independent review, a final set of 9 studies met all eligibility criteria and were included in the analysis [24–32]. A flow diagram detailing the process of study selection and inclusion is provided in Fig 1. Although some of the included studies did not explicitly use the term “cognitive frailty,” study eligibility was determined by the core conceptual features, specifically the simultaneous presence of physical frailty/prefrailty and mild cognitive impairment, without the presence of dementia. Accordingly, all studies reporting such coexisting conditions were considered eligible for inclusion, regardless of whether they specifically used the label “cognitive frailty.” This approach ensured that all relevant evidence was captured, enhancing the comprehensiveness of the review.

**Fig 1:**
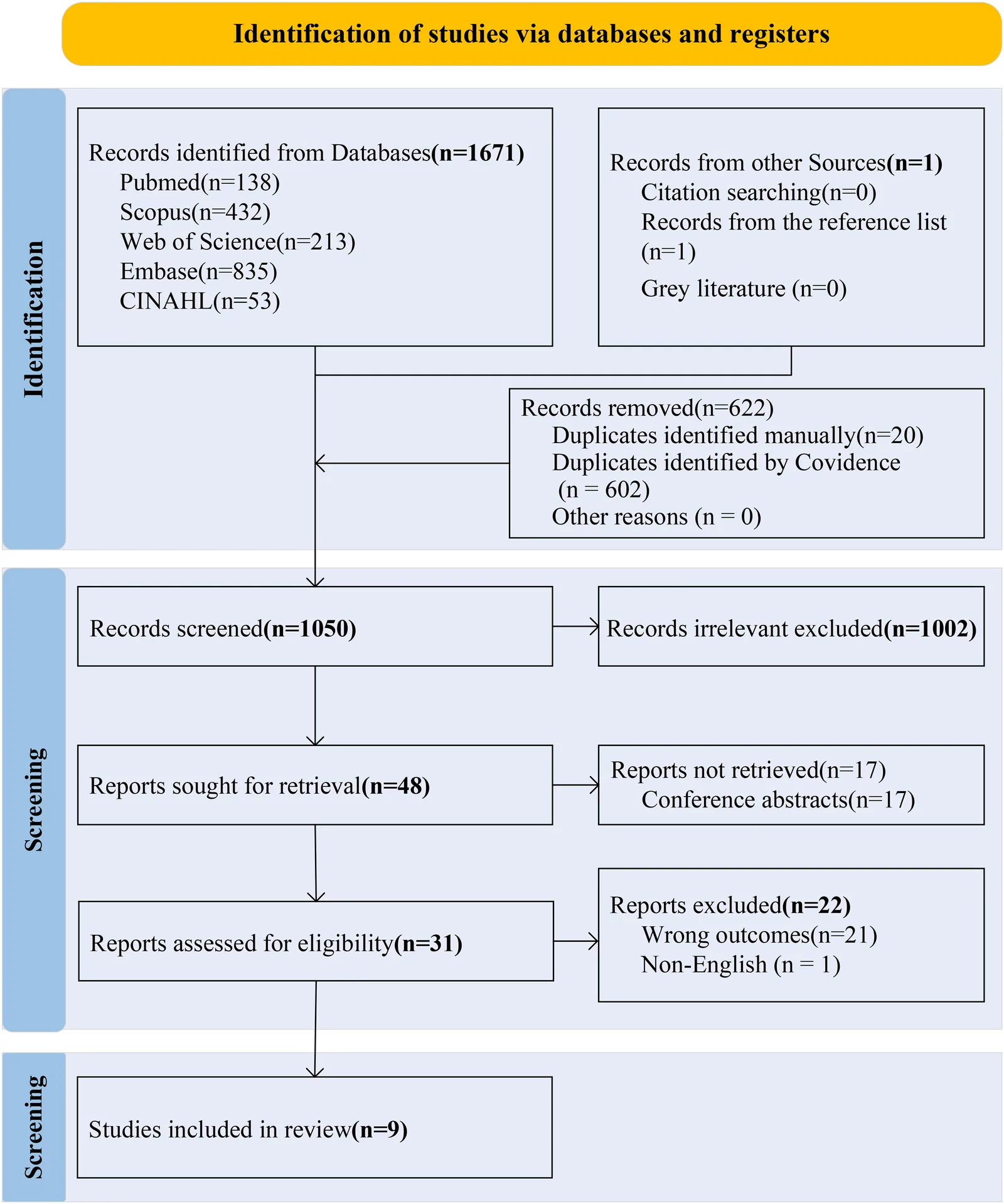
PRISMA flow diagram of the study selection process.

### 3.5 Quality appraisal

Two reviewers independently assessed the methodological quality of each included study using the Crowe Critical Appraisal Tool (CCAT) [33]. This tool was selected because it accommodates diverse research designs and yields a standardized quality score. Although critical appraisal is not mandatory in scoping reviews, quality assessment was conducted in this study for descriptive purposes only-to characterise the methodological quality of the included literature-rather than as an inclusion/exclusion criterion. This approach aims to provide contextual information on the robustness of the evidence and to support cautious interpretation of the findings. When differences of opinion occurred between the two reviewers during the quality assessment stage, they were addressed by seeking input from the third reviewer.

### 3.6 Data abstraction and synthesis

Two reviewers extracted data from the studies using a purpose-built Excel template developed in accordance with JBI guidance. The extracted information encompassed authors, year of publication, country, study type, sample size, participant age, participant sex, assessment instruments, prevalence estimates, and reported factors or outcomes. These data were then synthesised using a descriptive narrative approach, which integrated study characteristics, prevalence findings, assessment methods, risk factors, and prognostic associations. The PRISMA-ScR checklist (S2 Table) guided the reporting of this review.

## 4. Results

### 4.1 Characteristics of the included studies

This review included 9 studies [24–32] published between 2019 and 2026. Overall, the included studies demonstrated diversity in study design, geographical origin, and participant characteristics. In terms of study design and geographical distribution, the included studies were predominantly conducted in Europe, including Spain [24,26,27,32] (n = 4), the United Kingdom [25,29] (n = 2), Italy [28] (n = 1), and Poland [30] (n = 1). Additionally, one study from China [31] was also included. Regarding study design, one was observational [28] and the remaining eight were prospective observational [24–27,29–32]. In terms of center involvement, three were multi-center [25,26,29] and six were single-center [24,27,28,30–32]. Regarding the timing of assessment, all nine studies measured cognitive frailty at a single time point during hospitalisation; none provided longitudinal data to track changes over time.

Across studies, participant characteristics varied substantially. Sample sizes ranged from 196 to 547 individuals, with the proportion of women varying from 33.2% to 57.5%, and mean participant ages spanned from 71 to 83 years. Only four studies covered both subtypes of ACS (STEMI and NSTE-ACS) [27,30–32], among these, one study reported that NSTE-ACS accounted for as high as 94.5% [31], while the remaining five studies focused solely on a single subtype [24–26,28,29]. Among these five, two were on STEMI [24,28] and three on NSTE-ACS [25,26,29]. All included studies demonstrated generally high methodological quality, with CCAT scores ranging from 75 to 98 out of 100. Detailed scoring information and study characteristics are provided in Supplementary S3 Table and Table 3, respectively.

**Table 3:** Characteristics of the included studies.

Article	CCAT	Nation	Study type	Sample Size	Age	Sex	Disease Diagnosis
Elena Calvo (2019) [24]	88	Spain	A prospective observational single-center study	259	≥75(mean age:83)	Male(57.9%) and female(42.1%)	STEMI
Sophie Z. Gu (2019) [25]	90	UK	A multi-center prospective cohort study	271	≥65(Mean age:80)	Male(62.4%) and female(37.6%)	NSTEACS
Oriol Rodríguez-Queraltó MD(2020) [26]	85	Spain	A multi-center prospective cohort study	498	≥80(Mean age:84)	Male(61.6%) and female(38.4%)	NSTEACS
Juan Sanchis (2021) [27]	88	Spain	A prospective single-center cohort study	342	≥65(Mean age:77)	Male(57%)and female(43%)	ACS(STEMI, NSTEACS)
Pasquale Mone (2022) [28]	75	Italy	An single-center observational study	301	≥65(Mean age:74)	Male(42.5%) and female(57.5%)	STEMI
Valerie Josephine Dirjayanto (2024) [29]	93	UK	A multi-centre prospective observational study	239	≥75 (Median: 80)	Male (62.3%)and female(37.7%)	NSTEACS
Radosław Wontor (2024) [30]	78	Poland	A prospective observational single-center study	196	≥65(Mean age:74)	Male(60.7%) and female(39.3%)	ACS(STEMI:50%NSTEACS:50%)
Shan Wang (2025) [31]	93	China	A prospective observational single-center study	547	≥65(Mean age:71)	Male(57.8%) and female(42.2%)	ACS (STEMI:5.5%; NSTEACS:94.5%)
Ana Isabel Molina-Ramos (2026) [32]	98	Spain	A prospective observational single-centre study	211	≥70(Mean age:77.5)	Male(66.8%) and female(33.2%)	ACS(STEMI:37%NSTEACS:63%)

### 4.2 Prevalence of cognitive frailty in ACS patients

Seven of the nine reviewed investigations reported prevalence rates for cognitive frailty among individuals with ACS. Although the subjects were all elderly individuals aged over 65 years and were hospitalized patients diagnosed with ACS, the prevalence of cognitive frailty in ACS patients varied due to differences in measurement tools and population characteristics. The overall prevalence ranged from 8% to 55% [24–26,29–32]. When stratified by ACS subtype, the prevalence of cognitive frailty in patients with NSTE-ACS ranged from 23% to 40% [25,26,29], whereas a markedly lower prevalence of 8% [24] was reported in patients with STEMI. However, comparisons across ACS subtypes were limited by the small number of studies including both groups.

Marked differences were also observed according to the combinations of frailty and cognitive assessment tools employed. Different assessment tools showed varying detection rates for cognitive frailty: Studies using the Frailty Phenotype combined with the Montreal Cognitive Assessment (MoCA) reported prevalence rates of 39% [25] and 40% [29], respectively. In contrast, studies applying the FRAIL scale together with the Short Portable Mental Status Questionnaire (SPMSQ) reported substantially lower prevalence estimates, ranging from 8% to 23% [24,26,32]. Intermediate prevalence of 14% was observed when the FRAIL scale was combined with the Mini-Mental State Examination (MMSE) [31], while the highest prevalence of 55% was reported in a study using the Tilburg Frailty Indicator combined with the MMSE [30].

Overall, the available evidence suggests that the estimated prevalence of cognitive frailty in ACS patients is highly sensitive to the choice of assessment instruments and diagnostic thresholds. Importantly, the identified studies primarily assessed cognitive frailty at the time of ACS diagnosis, with no longitudinal data available on its incidence or progression over time. Detailed prevalence estimates are summarised in Table 4.

**Table 4:** Summary table of cognitive frailty influencing factors and outcomes in ACS patients.

Article	Diagnostic criteria for cognitive frailty			Timepoint measured	Main findings Detection rate/Influencing factors/Outcomes
	Frailty/Prefrailty	Cognitive impairment	Assessment methods		
	Tools	Tools			
Elena Calvo (2019) [24]	The FRAIL scale	SPMSQ	Face to face	Inhospital	8%
Sophie Z. Gu (2019) [25]	Frailty Phenotype	MoCA	Face to face	Inhospital	39%
Oriol Rodríguez-Queraltó MD (2020) [26]	The FRAIL scale	SPMSQ	Face to face	Inhospital	23%
Juan Sanchis (2021) [27]	Frailty Phenotype	SPMSQ	Face to face	Inhospital	Outcomes:All-cause mortality (long-term),Readmission for acute myocardial infarction (long-term)
Pasquale Mone (2022) [28]	Frailty Phenotype	MMSE	Face to face	Inhospital	Influencing factors:age, diabetes,hypertension
Valerie Josephine Dirjayanto(2024) [29]	Frailty Phenotype	MoCA	Face to face	Inhospital	40%
Radosław Wontor (2024) [30]	The Tilburg Frailty Indicator	MMSE	Face to face	Inhospital	55%
					Influencing factors:Age,Hypertension
					Outcomes:Early (hospital) complications,Hospital stay, Major adverse cardiovascular and cerebrovascular events at 6 Months (Recurrent ACS; Recurrent revascularisation; Stroke; Death)
Shan Wang (2025) [31]	The FRAIL Scale	MMSE	Face to face	Inhospital	14%
					Influencing factors:Age,Education, Systolic Blood Pressure,Glucose level, Charlson Comorbidity Index,Malnutrition
					Outcomes: In-hospital major adverse cardiac events(No clinical significance)
Ana Isabel Molina-Ramos(2026) [32]	The FRAIL Scale	SPMSQ	Face to face	Inhospital	10.4%
					Influencing factors:Angiographic variables:Culprit lesion calcification, Diffuse culprit disease, Single-vessel disease, Thrombus in non-culprit lesions.
					Outcomes:30-day early adverse clinical events(infection, stroke/TIA, repeat ACS/revascularization, AKI, and death)

### 4.3 Assessment tools for cognitive frailty in patients with ACS

The assessment of cognitive frailty used a combined approach to evaluate frailty and cognitive impairment, all conducted face-to-face. However, substantial heterogeneity was observed in both the choice of instruments and the operational definitions applied. Frailty was most commonly assessed using the FRAIL scale, the Frailty Phenotype, or the Tilburg Frailty Indicator, while cognitive impairment was evaluated using the Short Portable Mental Status Questionnaire, the Mini-Mental State Examination, or the Montreal Cognitive Assessment. The most frequently applied diagnostic combinations was the FRAIL scale with the SPMSQ [24,26,32]. Only one study employed the Tilburg Frailty Indicator in combination with the MMSE [30].

Four of the included studies [24,27,28,30] included only frailty in their assessment, excluding pre-frailty, which may partly explain their lower prevalence estimates [24]. Notably, the study by Wontor et al. from Poland [30] also assessed only frailty yet reported a prevalence of 55%. This suggests that, beyond the operational definition itself, population characteristics and assessment tools may also influence prevalence estimates. Table 5 provides a detailed summary of the main frailty and cognitive impairment assessment tools used in each study.

**Table 5:** Cognitive impairment diagnostic tools.

Frailty/prefrailty				Cognitive impairment				
Tools	items	Components	Rating criteria	Tools	Items	Multiple language versions	Components	Score range (abnormal-normal)
The FRAIL scale	5	Fatigue,Resistance,Ambulation,Illness,Loss of Weight	Frailty≥3items;Pre-frail:1–2items;Robust = 0 items	SPMSQ/Pfeiffer test	10		Attention,Memory,Orientation	10−0
Frailty Phenotype	5	Weight loss,low physical activity,exhaustion, slowness,weakness	Frailty≥3items;Pre-frail:1–2items;Robust = none	MoCA	11	√	Attention,Language,Memory,Orientation,Concentration,Executive function,Visuospatial skill	0-30
The Tilburg Frailty Indicator	15	Self-reported in 3 domains:physical,psychological and social	Frailty = scores≥5	MMSE	11	√	Attention,Language,Memory,Orientation	0-30

### 4.4 Influencing factors of cognitive frailty in patients with ACS

The factors linked to cognitive frailty in people with ACS can be put into three main groups: demographic and lifestyle attributes, physical health-related factors, and disease-related clinical indicators. Among demographic and lifestyle factors, older age [28,30,31] and lower educational level [31] were reported to be associated with cognitive frailty. Physical condition-related factors associated with cognitive frailty included a higher Charlson Comorbidity Index [31] and indicators of malnutrition [31]. Several disease-related factors were also reported to be associated with cognitive frailty, elevated systolic blood pressure [31] and hypertension [28,30] were each reported as risk factors for cognitive frailty, similarly, higher glucose levels [31] and a diagnosis of diabetes [28] were associated with cognitive frailty. One study [32] explored angiographic characteristics and found that culprit lesion calcification, diffuse culprit disease, single-vessel disease, and thrombus in non-culprit lesions were associated with cognitive frailty.

### 4.5 Outcomes of cognitive frailty in patients with ACS

Cognitive frailty was found to be a key risk factor for the poor prognosis of ACS patients, with those suffering from cognitive frailty experiencing significantly worse clinical outcomes than those with normal cognitive function. Cognitive frailty was consistently associated with poorer clinical outcomes in patients with ACS compared with those without cognitive impairment or frailty. Both short-term and long-term adverse outcomes were reported. Two studies examined medium-to long-term outcomes. One found that the composite rate of major adverse cardiovascular and cerebrovascular events at 6 months-comprising recurrent ACS, revascularisation, stroke, and death-was also higher, but no significant differences were observed for individual events [30]. Another study suggested that cognitive frailty was associated with elevated long-term mortality and readmission rates following ACS [27].

In terms of short-term prognosis, patients with ACS and concomitant cognitive frailty had a higher risk of early in-hospital complications [30], prolonged hospital stay [30], and an elevated composite incidence of 30-day early adverse clinical events [32], including infection, stroke/transient ischaemic attack, recurrent ACS/revascularisation, acute kidney injury, and death. However, none of the individual events reached statistical significance. Of note, one study reported that the risk of in-hospital major adverse cardiovascular events (MACE) was not clinically meaningful in patients with cognitive frailty [31].

## 5. Discussion

### 5.1 High prevalence and the need for a broader focus

A meta‑analysis reported a pooled cognitive frailty prevalence of 16% for individuals with mild cognitive impairment and concurrent frailty; the pooled prevalence dropped to 6% after excluding participants with pre‑frailty [34]. In contrast, the prevalence observed in the present review among patients with ACS may suggest that ACS itself is associated with an increased risk of cognitive frailty. This observation is consistent with the shared pathophysiological mechanisms (e.g., inflammation, oxidative stress, and neuroendocrine dysregulation) known to underlie both cognitive frailty and ACS [35]. Beyond overall prevalence estimates, one study reported higher frailty rates among patients with ST‑elevation myocardial infarction relative to those with non‑ST‑elevation myocardial infarction, a pattern consistent with our findings [36].

Another descriptive research gap is that all included studies assessed cognitive frailty only during hospitalization, without assessments before admission or after discharge. Consequently, the current literature does not capture pre‑existing cognitive frailty, changes during recovery after ACS, or trajectories in community settings. It remains unclear whether the observed cognitive frailty represents a pre‑existing chronic condition, a transient phenomenon triggered by the acute cardiac event, or a combination of both. Future studies that include assessments outside the hospital setting are needed to comprehensively describe the continuum of cognitive frailty in this population. The incidence of acute myocardial infarction MI has climbed steadily among middle-aged patients in the past several years [37], and its long-term consequences are often more severe in younger individuals [38], leading to a greater loss of life expectancy and significant healthcare costs [39]. While cognitive frailty has traditionally been viewed as a geriatric syndrome, new findings show that younger people can also be affected. This is especially true for those with long‑term or serious health conditions [40,41]. However, most existing studies remain focused on older adults, and the impact on younger patients is often overlooked. Future research should therefore broaden its scope to include ACS patients across all age groups.

### 5.2 Standardizing and enhancing methods to assess cognitive frailty in individuals with ACS

The observed variability in detection rates across studies likely reflects a lack of methodological consistency. Such inconsistency arises from divergent operational definitions of cognitive frailty, as well as from variations in diagnostic criteria and assessment instruments.Numerous instruments are available for cognitive assessment, yet their performance varies across populations due to factors such as educational background, language, culture, and the use of different cutoff scores. These variations have prompted some investigators to note the scarcity of well-validated cognitive measures with broad applicability. Despite these limitations, the use of a screening tool remains essential for detecting cognitive impairment [42]. Despite the original operationalization of cognitive frailty incorporating the CDR, the majority of reviewed studies relied on global cognitive screening instruments, notably the SPMSQ, to assess cognitive impairment.

Similarly, multiple instruments are available for frailty screening in clinical practice, but no consensus has been reached on their prognostic value, nor has a universally accepted reference standard been established for routine use [43]. Researchers commonly adopt two distinct conceptual frameworks for frailty: the syndromic model, operationalized via tools such as the Fried criteria, and the deficit‑accumulation model, represented by the Frailty Index (FI), which views frailty as a continuous spectrum. Some studies have reported that the FI predicts mortality with greater accuracy than the frailty phenotype; however, an important caveat should be noted: the inclusion of physical performance tests does not consistently enhance its predictive performance, and self-reported simple questions may sometimes yield comparable results [44].

Separate generic scales for physical frailty and cognitive function are presently the primary tools used to identify cognitive frailty among individuals with ACS, with the FRAIL scale with the SPMSQ combination being the most frequently employed. No validated instrument is specifically available for this population; consequently, diagnosis currently depends on concurrent impairment identified by both scales. This approach, however, introduces practical challenges: it complicates clinical decision‑making, requires clinicians to master multiple scales, and prolongs assessment time. These practical drawbacks underscore the need for further research to establish precise definitions and standardized assessment protocols for cognitive frailty in ACS patients. A notable gap in current practice is the limited attention given to assessing specific cognitive domains in ACS patients, despite the widespread administration of global cognitive evaluations. Comprehensive screening tools differ in their sensitivity to various cognitive functions. This highlights the need for more targeted, domain-specific instruments. The trajectory of decline across these domains remains poorly characterized, as does the timing of such changes during the course of cognitive impairment. We need a more detailed way to assess cognition. This approach can help us better understand how thinking changes in these patients. With that knowledge, we can spot problems earlier. We can also design more focused treatments.

### 5.3 Discussion on the influencing factors of cognitive frailty in ACS patients

This scoping review summarized the factors associated with cognitive frailty in patients with acute coronary syndrome ACS, which could be categorized into three major groups: demographic and lifestyle factors, physical health-related factors, and disease-related clinical indicators.

At the demographic and lifestyle level, older age and lower educational level were associated with cognitive frailty in ACS patients. Aging brings a series of biological changes that drive the development of frailty as a geriatric syndrome; meanwhile, cognitive impairment is also commonly seen in older adults. These two geriatric syndromes often co-occur and interact, potentially creating a downward spiral of deterioration [45]. The bidirectional relationship may be partially explained by shared pathological pathways: with increasing age, Alzheimer’s disease-like biomarkers, including cerebrospinal fluid markers and amyloid signals on PET-CT, accumulate and participate in the pathophysiology of cognitive frailty [46].

A high educational level was identified as a protective factor against cognitive frailty in Chinese ACS patients, which is consistent with the findings of Lee et al. [47]. Educational level is an important proxy for cognitive reserve, and multiple studies have confirmed that it reduces the risk of physical frailty and cognitive frailty in older adults [48,49]. Education may moderate the adverse effects of physical frailty on cognitive function, and higher educational attainment can buffer the cognitive decline induced by physical frailty. Its protective effects may also be mediated through socioeconomic pathways: individuals with higher education are more likely to adopt healthy lifestyles, have better access to healthcare resources [50–52], and possess better health knowledge and self-management abilities [53,54]. Existing evidence suggests that education is a marker of life-course cognitive reserve, and this protective benefit mainly derives from advantages accumulated earlier in life, rather than solely from cognitive activities in old age.

This review found that physical health-related factors associated with cognitive frailty included higher Charlson Comorbidity Index scores and indicators of malnutrition. Elevated Charlson Comorbidity Index scores, reflecting multimorbidity burden, were associated with cognitive frailty, consistent with previous studies [47,55]. Multimorbidity is highly prevalent in older adults and increases the risk of physical frailty and cognitive decline [56,57]. The accumulation of multiple chronic diseases represents widespread multi-system aging, depleting physiological reserve and accelerating frailty progression. Malnutrition was also significantly associated with cognitive frailty, in line with a meta-analysis [58]. Malnutrition is a risk factor for both physical frailty and cognitive impairment [59]; conversely, physical frailty or cognitive decline can cause decreased appetite and reduced self‑care ability in older adults, further exacerbating malnutrition and forming a vicious cycle [60]. Biologically, malnutrition can directly damage brain structure and hinder neuronal regeneration, thereby inducing cognitive dysfunction [58]. In summary, multimorbidity and malnutrition together reflect reduced physiological resilience, which may increase susceptibility to cognitive frailty in ACS patients.

Regarding disease-related clinical indicators, this review suggested that elevated systolic blood pressure, hypertension, elevated glucose levels, and diabetes were risk factors for cognitive frailty in ACS patients. Chronic hypertension can cause sustained, progressive damage to the nervous system [61]. When vascular risk factors such as hypertension and diabetes coexist with cardiovascular, cerebrovascular, and other chronic conditions, they may exert synergistic detrimental effects, accelerating the progression of cognitive frailty. Notably, one included study investigating coronary angiographic characteristics in patients with coronary artery disease found that culprit lesion calcification, diffuse culprit disease, single-vessel disease, and thrombus in non-culprit lesions were all associated with cognitive frailty, providing new clues for elucidating the vascular pathological basis of cognitive frailty. Studies in community-dwelling older adults can offer references for mechanistic interpretation: Park et al. found that vascular dysfunction, manifested as increased arterial stiffness and endothelial dysfunction, might serve as an intermediary linking frailty and cardiovascular disease [62]. Akaida et al. further observed that when physical frailty coexists with cognitive impairment-that is, in a state of cognitive frailty-the association with vascular dysfunction becomes even more pronounced [36]. However, both of the above studies are cross-sectional in design and cannot establish a causal temporal relationship between vascular dysfunction and cognitive frailty. Future longitudinal cohort studies in ACS populations are needed to clarify whether vascular dysfunction is an independent risk factor for the development of cognitive frailty and to further elucidate the underlying molecular pathological pathways.

### 5.4 Interventions to improve adverse outcomes in ACS cognitive frailty

Studies included in this scoping review consistently demonstrated that ACS patients with cognitive frailty experienced higher frequencies of composite endpoints, including in‑hospital complications, prolonged hospital stay, 30-day adverse events, and major cardiovascular and cerebrovascular events within six-month follow-up, compared with ACS patients without cognitive frailty. Nevertheless, when individual components of these composite endpoints (e.g., recurrent ACS alone, stroke, revascularization, or mortality) were analysed separately across these studies, most reported no statistically significant between-group differences. In addition, one study indicated that no clinically meaningful elevation in in-hospital major adverse cardiovascular event rates was observed among patients with cognitive frailty. Regarding mortality, which is commonly incorporated into composite adverse-event endpoints, only a single study reported an independent association between long-term mortality and cognitive frailty. Such discrepancies between composite endpoints and individual endpoints may be partially attributable to insufficient statistical power stemming from relatively small sample sizes and low event rates for specific outcomes, rather than a true absence of effect. The need for future longitudinal cohort studies with rigorous design, larger sample sizes, and standardised assessment protocols is evident-such studies would systematically examine the associations between these specific endpoints and provide more robust descriptive evidence for this clinically high-risk subgroup.

One characteristic of cognitive frailty is its reversibility [63]. Importantly, our review identified no intervention studies specifically designed for ACS patients with concomitant cognitive frailty. Although multi-component interventions (e.g., physical exercise, nutritional support, cognitive training) have been explored in general geriatric populations with cognitive frailty [64], their transferability to the acute cardiac setting has not been empirically tested. This finding does not imply that such interventions are ineffective, but rather highlights a clear evidence gap specific to this distinct and clinically vulnerable subgroup. Although most of the retrieved studies were prospective in design, they primarily examined baseline cognitive frailty as a predictor of subsequent adverse outcomes, without repeated assessments of cognitive frailty status over time. Consequently, the current evidence does not capture the dynamic evolution of cognitive frailty or its temporal relationship with changes in clinical status during the post‑ACS course. This analytical approach, rather than the study design per se, limits the ability to describe trajectories of cognitive frailty or to characterise its bidirectional interactions with other clinical factors. Future longitudinal investigations that incorporate serial measurements of both cognitive and physical frailty, along with harmonised diagnostic criteria, would help delineate the natural history of this condition in the ACS population and identify potentially modifiable factors that could inform the design of patient‑centred care strategies.

### 5.5 Strengths and limitations

To the best of our knowledge, this represents the first scoping review to focus specifically on cognitive frailty in patients with ACS. Additional strengths include the rigorous methodology of our review process, which involved dual independent screening, quality assessment, and data extraction. Finally, the comprehensive search strategy helped ensure that potentially relevant evidence was captured for synthesis.

This review has several limitations that should be acknowledged. First, the literature search was restricted to English-language databases, potentially omitting relevant evidence published in other languages. Second, the small final sample of only nine included studies limits the breadth of the evidence and the robustness of the conclusions. Finally, The included studies exhibited significant heterogeneity in terms of assessment tools, diagnostic criteria, and population characteristics. This variability led to a wide range of reported prevalence, meaningful direct comparisons between studies and combined data analyses proved challenging.

## 6. Conclusion

This review indicate that cognitive frailty is commonly reported in patients with ACS, with prevalence estimates varying considerably across studies. and that multiple investigations have noted an association with adverse clinical outcomes. As a critical determinant of long-term prognosis, this condition warrants greater attention in cardiovascular practice. The development of cognitive frailty is influenced by multiple factors, reflecting complex underlying pathophysiological mechanisms. However, existing studies on ACS-related cognitive frailty exhibit substantial heterogeneity in assessment tools and diagnostic criteria, with no standardized approach, hindering direct comparison and synthesis of findings. Notably, all included studies assessed cognitive frailty exclusively during hospitalisation, without pre‑admission or post‑discharge measurements, indicating a descriptive gap regarding pre‑existing cognitive status, recovery‑phase changes, and community trajectories. Furthermore, although most studies were prospective, they relied solely on baseline measurements and lacked repeated assessments, leaving the dynamic evolution of cognitive frailty over the ACS course poorly characterised. Future efforts should focus on high-quality prospective longitudinal studies, the development of rapid screening instruments suitable for cardiovascular settings, and the implementation of multidisciplinary intervention strategies. These approaches are essential to comprehensively understand the pathogenesis, risk factors, and clinical outcomes of cognitive impairment in ACS patients, and to provide robust evidence for improving cardiovascular prognosis in this high-risk population.

## Supporting information

S1 Table Search strategy used for each of the databases. (DOCX)

S2 Table Preferred Reporting Items for Systematic reviews and Meta-Analyses extension for Scoping Reviews (PRISMA-ScR) Checklist. (PDF)

S3 Table CCAT scoring criteria. (DOCX)
