# Supplementary material for: Cognitive frailty in patients with acute coronary syndromes: A scoping review
Source: PLoS One. 2026 Oct 30;21(10):e0361499. doi: 10.1371/journal.pone.0361499 (PMC13722203; doi:10.1371/journal.pone.0361499)
Supplement: Supplementary file 2 [file pone.0361499.s001.docx]

| **Pubmed (Search time:August 13, 2026)** | | |
| --- | --- | --- |
| **Search** | **Query** | **Results** |
| 1 | "Acute Coronary Syndrome"[Mesh] | 22951 |
| 2 | "Acute Coronary Syndromes" OR "heart attack" OR "Coronary Syndrome*, Acute" OR "Syndromes, Acute Coronary" OR "ACS" OR "ST Elevat* Myocardial Infarction" OR "ST Segment Elevation Myocardial Infarction" OR "STEMI" OR "Non-ST Elevated Myocardial Infarction" OR "Non ST Elevat* Myocardial Infarction" OR "NSTEMI" OR "Non-ST-Elevation Myocardial Infarction*" OR "Infarction*, Non-ST-Elevation Myocardial" OR "Myocardial Infarction*, Non-ST-Elevation" OR "Angina*, Unstable" OR "Unstable Angina*" OR "Angina at Rest" OR "Angina*, Preinfarction" OR "Preinfarction Angina*" OR "Angina Pectori*, Unstable" OR "Unstable Angina Pectori*" OR "Myocardial Pre-infarction Syndrome*" OR "Pre-infarction Syndrome*, Myocardial" OR "Syndrome*, Myocardial Pre-infarction" OR "Myocardial Infarction*" OR "Infarction*, Myocardial" OR "Heart Attack*" OR "Myocardial Infarct*" OR "Infarct*, Myocardial" OR "Cardiovascular Stroke*" OR "Stroke*, Cardiovascular" OR "Ischemias, Myocardial" OR "Ischemic Heart Disease" OR "Diseases, Ischemic Heart" OR "Artery Disease, Coronary" OR "Artery Diseases, Coronary" | 575396 |
| 3 | 1 OR 2 | 580990 |
| 4 | "cognit* frail*" OR ((frail* OR "Frailty Syndrome" OR "physical frailty") AND (Cogniti* OR "mild cognitive impairment*" OR "Cognitive Function*" OR "Function*, Cognitive" OR "Disorder*, Cognition" OR "mci" OR "cognitive decline*" OR "cognitive impairment*" OR "Cognition Disorder*" OR "Cognitive Dysfunctions" OR "Dysfunction, Cognitive" OR "Dysfunctions, Cognitive" OR "Cognitive Disorder*" OR "Impairment*, Cognitive" OR "Cognitive Impairment*, Mild" OR "Impairment*, Mild Cognitive" OR "Decline*, Cognitive" OR "cognitive defects" OR "cognitive deficiency" OR "cognitive deficit" OR "cognitive difficulties" OR "cognitive disability" OR "cognitive problems")) | 9315 |
| 5 | 3 AND 4 | 138 |
| **Embase (Search time:August 13, 2026)** | | |
| **Search** | **Query** | **Results** |
| 1 | 'acute coronary syndrome'/exp | 95436 |
| 2 | "Acute Coronary Syndromes" OR "heart attack" OR "Coronary Syndrome*, Acute" OR "Syndromes, Acute Coronary" OR "ACS" OR "ST Elevat* Myocardial Infarction" OR "ST Segment Elevation Myocardial Infarction" OR "STEMI" OR "Non-ST Elevated Myocardial Infarction" OR "Non ST Elevat* Myocardial Infarction" OR "NSTEMI" OR "Non-ST-Elevation Myocardial Infarction*" OR "Infarction*, Non-ST-Elevation Myocardial" OR "Myocardial Infarction*, Non-ST-Elevation" OR "Angina*, Unstable" OR "Unstable Angina*" OR "Angina at Rest" OR "Angina*, Preinfarction" OR "Preinfarction Angina*" OR "Angina Pectori*, Unstable" OR "Unstable Angina Pectori*" OR "Myocardial Pre-infarction Syndrome*" OR "Pre-infarction Syndrome*, Myocardial" OR "Syndrome*, Myocardial Pre-infarction" OR "Myocardial Infarction*" OR "Infarction*, Myocardial" OR "Heart Attack*" OR "Myocardial Infarct*" OR "Infarct*, Myocardial" OR "Cardiovascular Stroke*" OR "Stroke*, Cardiovascular" OR "Ischemias, Myocardial" OR "Ischemic Heart Disease" OR "Diseases, Ischemic Heart" OR "Artery Disease, Coronary" OR "Artery Diseases, Coronary" | 130,512 |
| 3 | 1 OR 2 | 131,149 |
| 4 | "cognit* frail*" OR ((frail* OR "Frailty Syndrome" OR "physical frailty") AND (Cogniti* OR "mild cognitive impairment*" OR "Cognitive Function*" OR "Function*, Cognitive" OR "Disorder*, Cognition" OR "mci" OR "cognitive decline*" OR "cognitive impairment*" OR "Cognition Disorder*" OR "Cognitive Dysfunctions" OR "Dysfunction, Cognitive" OR "Dysfunctions, Cognitive" OR "Cognitive Disorder*" OR "Impairment*, Cognitive" OR "Cognitive Impairment*, Mild" OR "Impairment*, Mild Cognitive" OR "Decline*, Cognitive" OR "cognitive defects" OR "cognitive deficiency" OR "cognitive deficit" OR "cognitive difficulties" OR "cognitive disability" OR "cognitive problems")) | 17607 |
| 5 | 3 AND 4 | 835 |
| **CINAHL Complete (Search time:August 13, 2026)** | | |
| **Search** | **Query** | **Results** |
| 1 | 'acute coronary syndrome'/exp | 8324 |
| 2 | "Acute Coronary Syndromes" OR "heart attack" OR "Coronary Syndrome*, Acute" OR "Syndromes, Acute Coronary" OR "ACS" OR "ST Elevat* Myocardial Infarction" OR "ST Segment Elevation Myocardial Infarction" OR "STEMI" OR "Non-ST Elevated Myocardial Infarction" OR "Non ST Elevat* Myocardial Infarction" OR "NSTEMI" OR "Non-ST-Elevation Myocardial Infarction*" OR "Infarction*, Non-ST-Elevation Myocardial" OR "Myocardial Infarction*, Non-ST-Elevation" OR "Angina*, Unstable" OR "Unstable Angina*" OR "Angina at Rest" OR "Angina*, Preinfarction" OR "Preinfarction Angina*" OR "Angina Pectori*, Unstable" OR "Unstable Angina Pectori*" OR "Myocardial Pre-infarction Syndrome*" OR "Pre-infarction Syndrome*, Myocardial" OR "Syndrome*, Myocardial Pre-infarction" OR "Myocardial Infarction*" OR "Infarction*, Myocardial" OR "Heart Attack*" OR "Myocardial Infarct*" OR "Infarct*, Myocardial" OR "Cardiovascular Stroke*" OR "Stroke*, Cardiovascular" OR "Ischemias, Myocardial" OR "Ischemic Heart Disease" OR "Diseases, Ischemic Heart" OR "Artery Disease, Coronary" OR "Artery Diseases, Coronary" | 130512 |
| 3 | 1 OR 2 | 1.31149 |
| 4 | "cognit* frail*" OR ((frail* OR "Frailty Syndrome" OR "physical frailty") AND (Cogniti* OR "mild cognitive impairment*" OR "Cognitive Function*" OR "Function*, Cognitive" OR "Disorder*, Cognition" OR "mci" OR "cognitive decline*" OR "cognitive impairment*" OR "Cognition Disorder*" OR "Cognitive Dysfunctions" OR "Dysfunction, Cognitive" OR "Dysfunctions, Cognitive" OR "Cognitive Disorder*" OR "Impairment*, Cognitive" OR "Cognitive Impairment*, Mild" OR "Impairment*, Mild Cognitive" OR "Decline*, Cognitive" OR "cognitive defects" OR "cognitive deficiency" OR "cognitive deficit" OR "cognitive difficulties" OR "cognitive disability" OR "cognitive problems")) | 4009 |
| 5 | 3 AND 4 | 53 |
| **Web of sciences (Search time:August 13, 2026)** | | |
| **Search** | **Query** | **Results** |
| 1 | "Acute Coronary Syndromes" OR "heart attack" OR "Coronary Syndrome*, Acute" OR "Syndromes, Acute Coronary" OR "ACS" OR "ST Elevat* Myocardial Infarction" OR "ST Segment Elevation Myocardial Infarction" OR "STEMI" OR "Non-ST Elevated Myocardial Infarction" OR "Non ST Elevat* Myocardial Infarction" OR "NSTEMI" OR "Non-ST-Elevation Myocardial Infarction*" OR "Infarction*, Non-ST-Elevation Myocardial" OR "Myocardial Infarction*, Non-ST-Elevation" OR "Angina*, Unstable" OR "Unstable Angina*" OR "Angina at Rest" OR "Angina*, Preinfarction" OR "Preinfarction Angina*" OR "Angina Pectori*, Unstable" OR "Unstable Angina Pectori*" OR "Myocardial Pre-infarction Syndrome*" OR "Pre-infarction Syndrome*, Myocardial" OR "Syndrome*, Myocardial Pre-infarction" OR "Myocardial Infarction*" OR "Infarction*, Myocardial" OR "Heart Attack*" OR "Myocardial Infarct*" OR "Infarct*, Myocardial" OR "Cardiovascular Stroke*" OR "Stroke*, Cardiovascular" OR "Ischemias, Myocardial" OR "Ischemic Heart Disease" OR "Diseases, Ischemic Heart" OR "Artery Disease, Coronary" OR "Artery Diseases, Coronary" | 945781 |
| 2 | "cognit* frail*" OR ((frail* OR "Frailty Syndrome" OR "physical frailty") AND (Cogniti* OR "mild cognitive impairment*" OR "Cognitive Function*" OR "Function*, Cognitive" OR "Disorder*, Cognition" OR "mci" OR "cognitive decline*" OR "cognitive impairment*" OR "Cognition Disorder*" OR "Cognitive Dysfunctions" OR "Dysfunction, Cognitive" OR "Dysfunctions, Cognitive" OR "Cognitive Disorder*" OR "Impairment*, Cognitive" OR "Cognitive Impairment*, Mild" OR "Impairment*, Mild Cognitive" OR "Decline*, Cognitive" OR "cognitive defects" OR "cognitive deficiency" OR "cognitive deficit" OR "cognitive difficulties" OR "cognitive disability" OR "cognitive problems")) | 10946 |
| 3 | 1 AND 2 | 213 |
| **Scopus (Search time:August 13, 2026)** | | |
| **Search** | **Query** | **Results** |
| 1 | "Acute Coronary Syndromes" OR "heart attack" OR "Coronary Syndrome*, Acute" OR "Syndromes, Acute Coronary" OR "ACS" OR "ST Elevat* Myocardial Infarction" OR "ST Segment Elevation Myocardial Infarction" OR "STEMI" OR "Non-ST Elevated Myocardial Infarction" OR "Non ST Elevat* Myocardial Infarction" OR "NSTEMI" OR "Non-ST-Elevation Myocardial Infarction*" OR "Infarction*, Non-ST-Elevation Myocardial" OR "Myocardial Infarction*, Non-ST-Elevation" OR "Angina*, Unstable" OR "Unstable Angina*" OR "Angina at Rest" OR "Angina*, Preinfarction" OR "Preinfarction Angina*" OR "Angina Pectori*, Unstable" OR "Unstable Angina Pectori*" OR "Myocardial Pre-infarction Syndrome*" OR "Pre-infarction Syndrome*, Myocardial" OR "Syndrome*, Myocardial Pre-infarction" OR "Myocardial Infarction*" OR "Infarction*, Myocardial" OR "Heart Attack*" OR "Myocardial Infarct*" OR "Infarct*, Myocardial" OR "Cardiovascular Stroke*" OR "Stroke*, Cardiovascular" OR "Ischemias, Myocardial" OR "Ischemic Heart Disease" OR "Diseases, Ischemic Heart" OR "Artery Disease, Coronary" OR "Artery Diseases, Coronary" | 610453 |
| 2 | "cognit* frail*" OR ((frail* OR "Frailty Syndrome" OR "physical frailty") AND (Cogniti* OR "mild cognitive impairment*" OR "Cognitive Function*" OR "Function*, Cognitive" OR "Disorder*, Cognition" OR "mci" OR "cognitive decline*" OR "cognitive impairment*" OR "Cognition Disorder*" OR "Cognitive Dysfunctions" OR "Dysfunction, Cognitive" OR "Dysfunctions, Cognitive" OR "Cognitive Disorder*" OR "Impairment*, Cognitive" OR "Cognitive Impairment*, Mild" OR "Impairment*, Mild Cognitive" OR "Decline*, Cognitive" OR "cognitive defects" OR "cognitive deficiency" OR "cognitive deficit" OR "cognitive difficulties" OR "cognitive disability" OR "cognitive problems")) | 13130 |
| 3 | 1 AND 2 | 432 |
