# Supplementary material for: Cognitive frailty in patients with acute coronary syndromes: A scoping review
Source: PLoS One. 2026 Oct 30;21(10):e0361499. doi: 10.1371/journal.pone.0361499 (PMC13722203; doi:10.1371/journal.pone.0361499)
Supplement: Supplementary file 3 [file pone.0361499.s003.docx]

| **Article** | **Preliminaries** | **Introduction** | **Design** | **Sampling** | **Data collection** | **Ethical matters** | **Results** | **Discussion** | **Raw total (/40)** | **Standardised score (/100)** |
| --- | --- | --- | --- | --- | --- | --- | --- | --- | --- | --- |
| Elena Calvo（2019）^[23]^ | 5 | 5 | 5 | 3 | 4 | 4 | 4 | 5 | 35 | 88 |
| Valerie Josephine Dirjayanto（2024）^[24]^ | 5 | 5 | 5 | 3 | 4 | 5 | 5 | 5 | 37 | 93 |
| Shan Wang（2025）^[25]^ | 5 | 5 | 4 | 3 | 4 | 5 | 5 | 5 | 37 | 93 |
| Sophie Z. Gu（2019）^[26]^ | 5 | 5 | 5 | 3 | 4 | 5 | 5 | 4 | 36 | 90 |
| Pasquale Mone  (2022)^[27]^ | 5 | 4 | 4 | 3 | 0 | 4 | 4 | 5 | 30 | 75 |
| Oriol Rodríguez-Queraltó MD(2020)^[28]^ | 5 | 5 | 3 | 4 | 4 | 5 | 3 | 5 | 34 | 85 |
| Juan Sanchis  (2021)^[29]^ | 5 | 5 | 5 | 3 | 5 | 4 | 4 | 4 | 35 | 88 |
| Radosław Wontor  (2024)^[30]^ | 5 | 5 | 4 | 2 | 3 | 4 | 3 | 5 | 31 | 78 |
| Ana Isabel Molina-Ramos(2026)^[]^ | 5 | 5 | 4 | 5 | 5 | 5 | 5 | 5 | 39 | 98 |
